# Supplementary material for: Mitochondrial Stress Links Environmental Triggers with Pro-Inflammatory Signaling in Crohn’s Disease
Source: Antioxidants (Basel). 2023 Dec 13;12(12):2105. doi: 10.3390/antiox12122105 (PMC10741078; doi:10.3390/antiox12122105)
Supplement: Supplementary file 1 [file antioxidants-12-02105-s001.zip › supplemental information File S1.pdf]

Replicate_1 (07-03-2022)

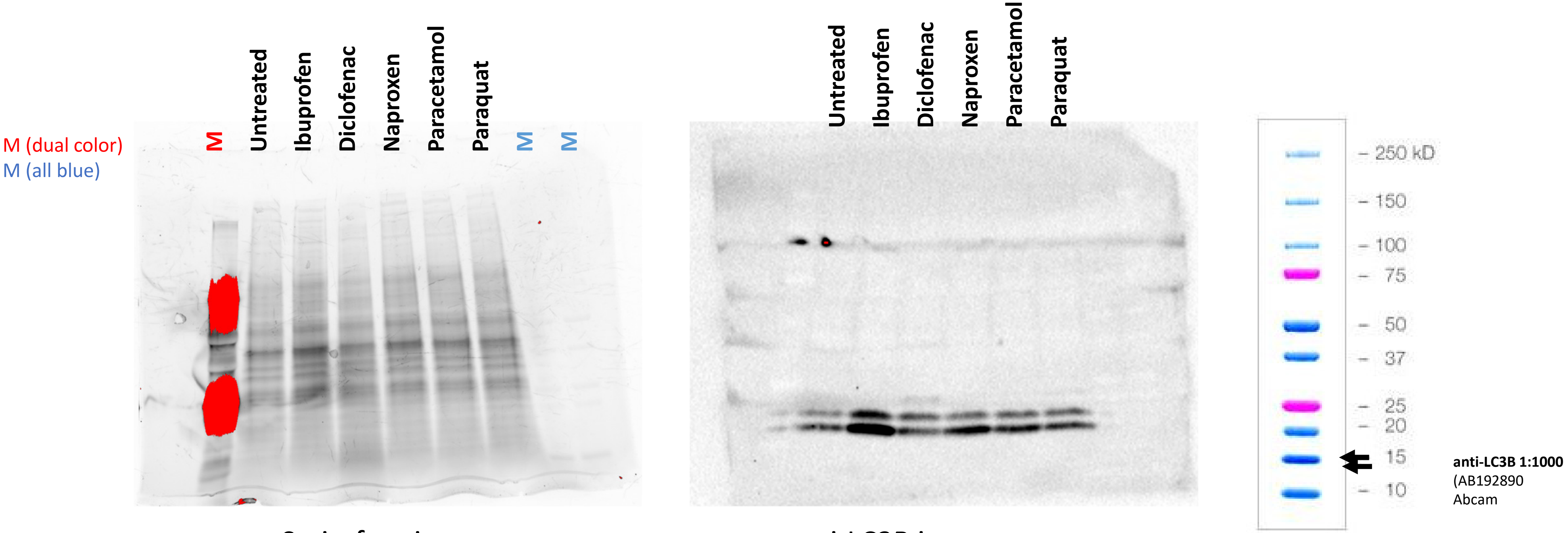

Stain-free image

anti-LC3B image

Replicate_2 (21-07-2022)

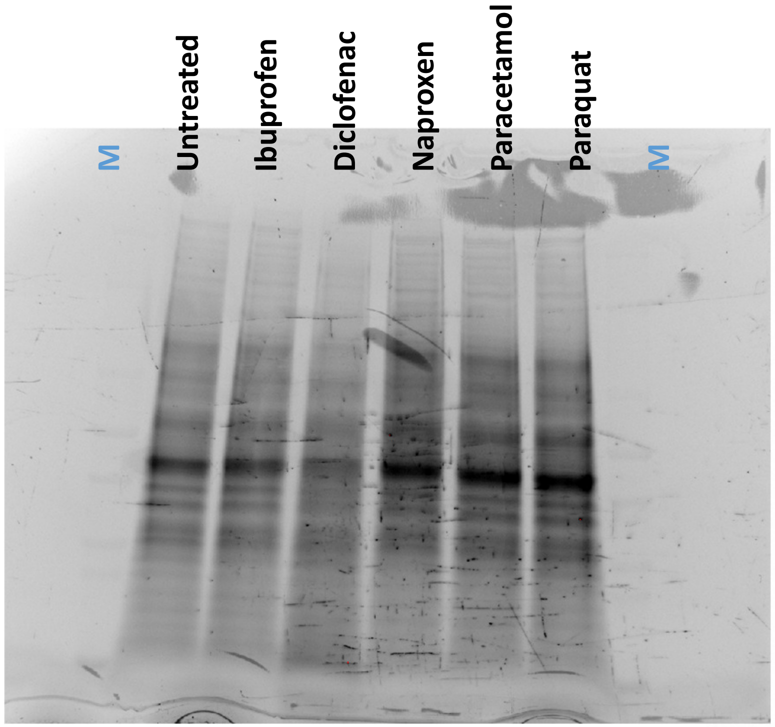

Stain-free image

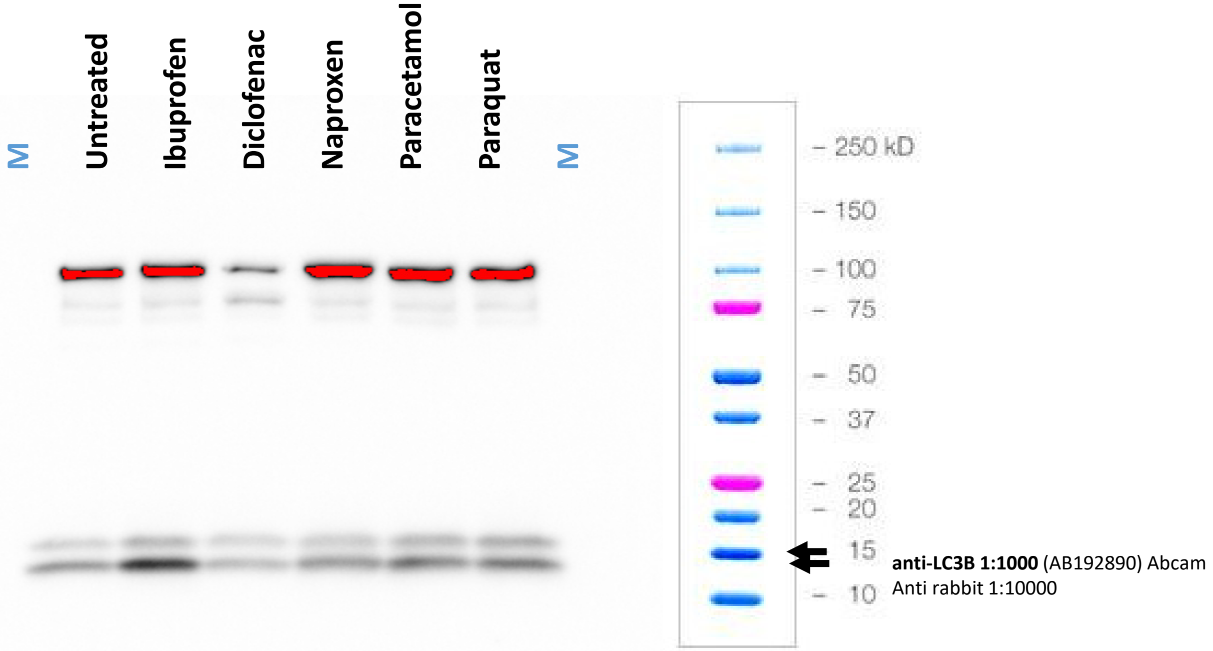

anti-LC3B image

Replicate_3
(09-01-2023)

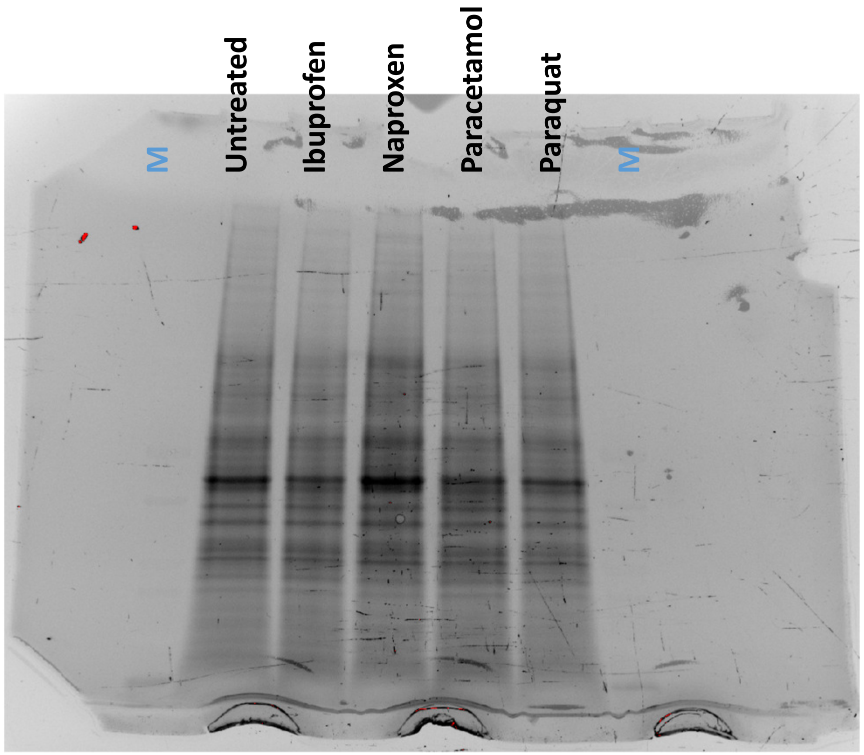

Stain-free image

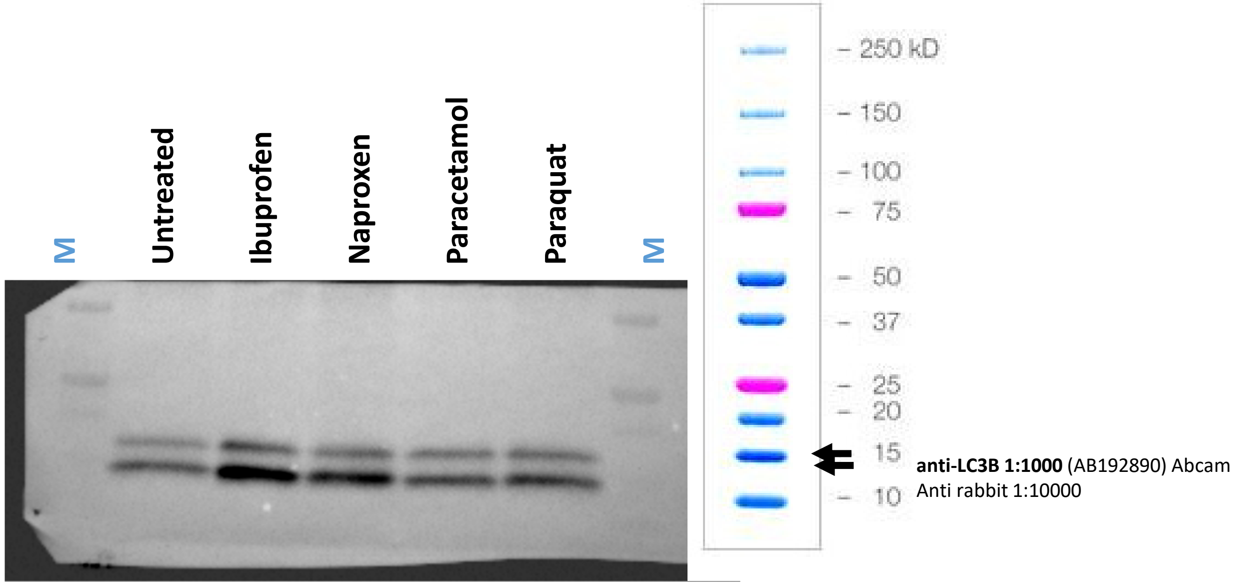

anti-LC3B image

Replicate_4
(27-11-2023)

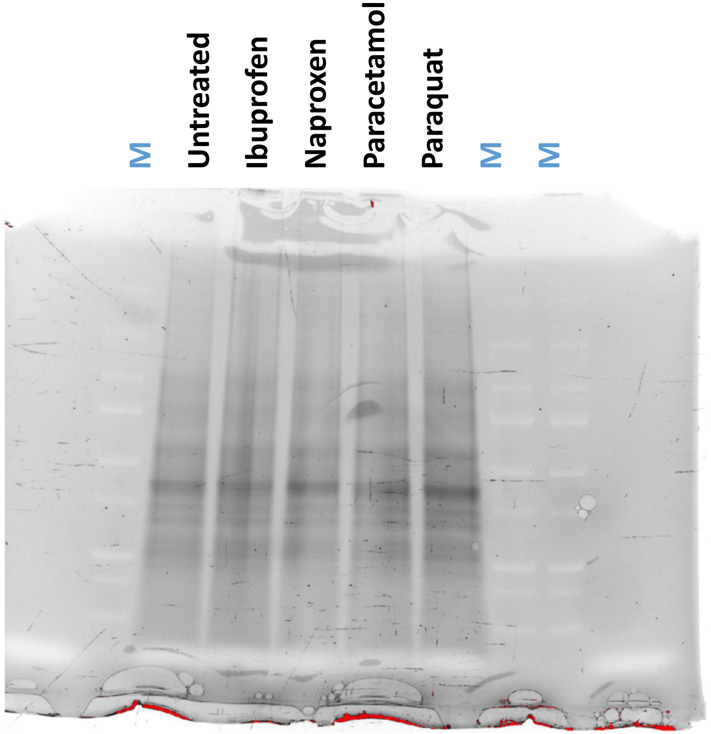

M (all blue) BioRad

Stain-free image

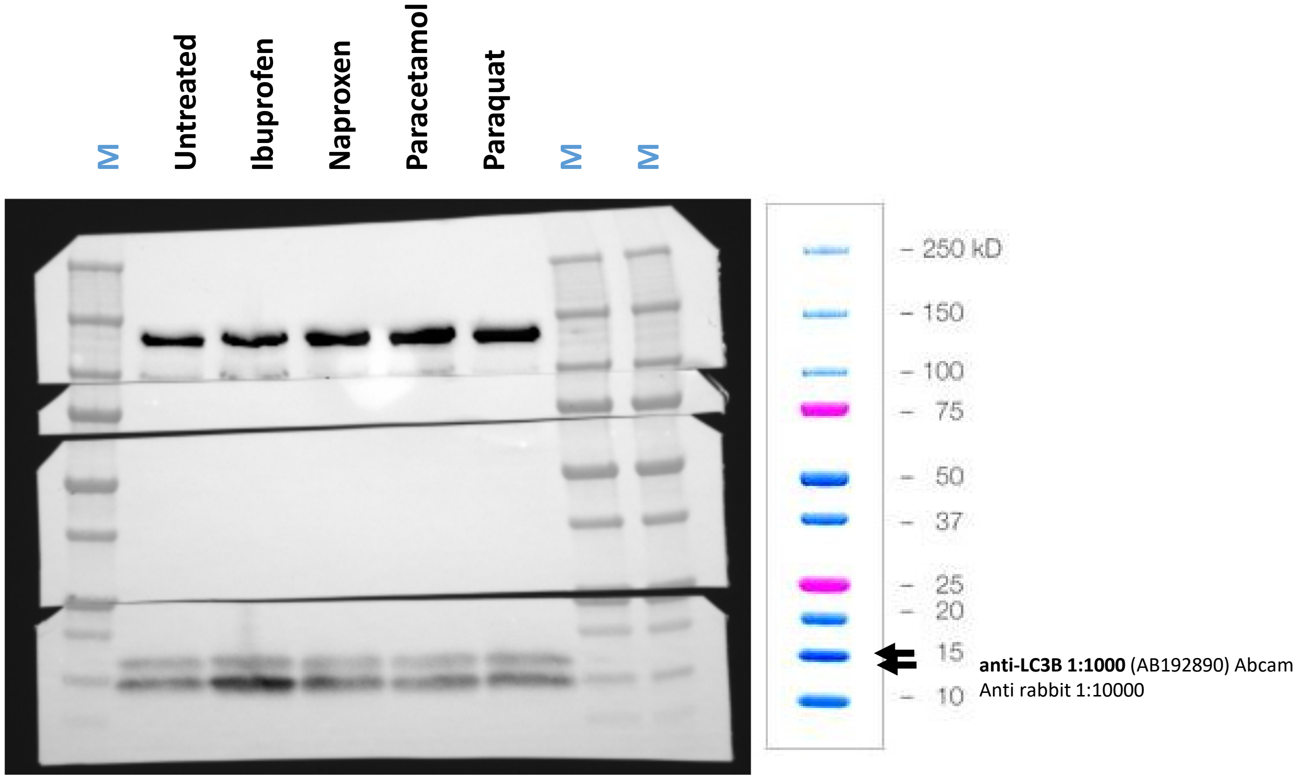

anti-LC3B image
