# Supplementary material for: Mitochondrial Stress Links Environmental Triggers with Pro-Inflammatory Signaling in Crohn’s Disease
Source: Antioxidants (Basel). 2023 Dec 13;12(12):2105. doi: 10.3390/antiox12122105 (PMC10741078; doi:10.3390/antiox12122105)
Supplement: Supplementary file 1 [file antioxidants-12-02105-s001.zip › supplementary tables.pdf]

Table S1. Demographic characteristic of subjects participating in the study. For gender, number of individuals from each gender and percentage is represented. For age at colonoscopy and disease duration, median and interquartile range (IQR, P25-P75) is represented. No significant difference was observed between different groups.

Baseline characteristics	Colonic organoids	
	HC	CD
Male/Female (%)	0/4 (0/100)	0/5 (0/100%)
Median (IQR) age at endoscopy (years)	48.5 (46.25-50.25)	42 (34-55)
Median (IQR) disease duration (years)	N/A	10 (3-11)

A. Demographic characteristics of subjects from whom colonic organoids were developed and used in the study.

Baseline characteristics	Healthy donors	Crohn´s disease		
		Remission	Low-to-moderate activity	Severe activity
Male/Female (%)	5/4 (55.6/44.4)	3/2 (60.0/40.0)	4/4 (50.0/50.0)	1/3 (25.0/75.0)
Median (IQR) age at endoscopy (years)	42.0 (32-52)	34 (33-46)	44 (39.75-46.5)	37.5 (29.25-46.25)
Median (IQR) disease duration (years)		10 (0-24)	4.5 (0-14)	9 (1.5-17.5)

B. Demographic characteristic of subjects from whom colonic biopsies were used in the study.

Table S2: Primers used for quantification of gene expression. Primer sequences (forward and reverse) for genes used in this study are represented in 5´ 3´direction. Main function is indicated.

Supplemental table 2. Primers used for RT-qPCR assays.

Gene	Forward (5`→ 3`)	Reverse (5`→ 3`)	Function
S18	AGAGAATTTGCCCTCCGACT	AACACTGGATTCCACCTTGG	Housekeeping
ATF4	TCAAACCTCATGGGTTCTCC	GTGTCATCCAACGTGGTCAG	UPR^{mt}
ATF5	TGGATCTCCTTCCCTCCTTT	GCTCTATCCTGTCGCCACTC	UPR^{mt}
CHOP	TGGAAGCCTGGTATGAGGAC	TGTGACCTCTGCTGGTTCTG	UPR^{mt}
GDF15	CTCCAGATTCCGAGAGTTGC	AGAGATACGCAGGTGCAGGT	Mitokine
FGF21	ACTCCAGTCCTCTCCTGCAA	ATCCTCCCTGATCTCCAGGT	Mitokine
PGC1α	CACCAGCCAACACTCAGCTA	GTGTGAGGAGGGTCATCGTT	Mitochondrial biogenesis
TRAP1	ATCCACCTGAAATCCGACTG	GCGACGTAGCGGTAGAACTC	Mitochondrial chaperone
LON	CGGGAAGATCATCCAGTGTT	ACGTCCAGGTAGTGGTCCAG	Mitochondrial protease
DRP1	GTGAACCCGTGGATGATAAA	GAAACCTCAGGCACAAATAAAG	Mitochondrial fission
FIS1	AGCGGGATTACGTCTTCTA	CCACGAGTCCATCTTTCTTC	Mitochondrial fission
OPA1	GCCACTTCCTGGGTCATTCC	CTGGCAGACCTCACAGGC	Mitochondrial fusion
NF-kB	CCTGGATGACTCTTGGGAAA	TCAGCCAGCTGTTTCATGTC	Pro-inflammatory transcription factor
OAS1	CGAAACCAACAGCAGTCCAA	TGGTTGATTTGCATGCGGAA	IFN pathway
STAT1	TGCAAAACCTTGCAGAACAG	GGGCATTCTGGGTAAGTTCA	IFN pathway

Table S3: Correlation between expression of assessed genes in colonic biopsies. For each pair of genes, Pearson´s R and p value (two tails) are shown. Color scale from red to green represents degree of correlation (red indicates Pearson´s r closer to 1 and green indicates Pearson´s r closer to 0).

		ATF4	ATF5	CHOP	GDF15	FGF21	PGC1α	TRAP1	DRP1	FIS1	OPA1	NFKB	OAS1	STAT1
**ATF4**	Pearson´s r		0.804	0.655	0.278	0.4	0.036	0.299	0.246	0.509	0.479	0.176	0.341	0.253
	p value		<0.001	<0.001	0.169	0.08	0.862	0.138	0.227	0.008	0.013	0.39	0.088	0.212
**ATF5**	Pearson´s r	0.804		0.514	-0.045	0.279	-0.048	0.24	0.373	0.709	0.56	0.488	0.257	0.417
	p value	<0.001		0.007	0.828	0.234	0.816	0.238	0.06	<0.001	0.003	0.011	0.205	0.034
**CHOP**	Pearson´s r	0.655	0.514		0.225	0.197	0.167	0.229	0.343	0.475	0.442	0.224	0.107	0.136
	p value	<0.001	0.007		0.27	0.405	0.416	0.261	0.086	0.014	0.024	0.271	0.601	0.508
**GDF15**	Pearson´s r	0.278	-0.045	0.225		-0.076	-0.099	0.155	-0.14	0.102	-0.069	-0.245	-0.028	-0.037
	p value	0.169	0.828	0.27		0.75	0.63	0.449	0.494	0.621	0.736	0.228	0.892	0.856
**FGF21**	Pearson´s r	0.4	0.279	0.197	-0.076		-0.217	-0.179	-0.115	0.314	-0.058	-0.11	0.123	0.065
	p value	0.08	0.234	0.405	0.75		0.359	0.45	0.63	0.177	0.809	0.646	0.604	0.785
**PGC1a**	Pearson´s r	0.036	-0.048	0.167	-0.099	-0.217		0.418	0.523	-0.04	0.479	0.437	0.428	-0.002
	p value	0.862	0.816	0.416	0.63	0.359		0.034	0.006	0.847	0.013	0.026	0.029	0.993
**TRAP1**	Pearson´s r	0.299	0.24	0.229	0.155	-0.179	0.418		0.44	0.316	0.305	0.375	0.633	0.62
	p value	0.138	0.238	0.261	0.449	0.45	0.034		0.024	0.116	0.13	0.059	0.001	0.001
**DRP1**	Pearson´s r	0.246	0.373	0.343	-0.14	-0.115	0.523	0.44		0.286	0.538	0.629	0.336	0.394
	p value	0.227	0.06	0.086	0.494	0.63	0.006	0.024		0.156	0.005	0.001	0.094	0.046
**FIS1**	Pearson´s r	0.509	0.709	0.475	0.102	0.314	-0.04	0.316	0.286		0.388	0.265	0.127	0.374
	p value	0.008	<0.001	0.014	0.621	0.177	0.847	0.116	0.156		0.05	0.191	0.536	0.06
**OPA1**	Pearson´s r	0.479	0.56	0.442	-0.069	-0.058	0.479	0.305	0.538	0.388		0.41	0.424	0.266
	p value	0.013	0.003	0.024	0.736	0.809	0.013	0.13	0.005	0.05		0.038	0.031	0.188
**NFKB**	Pearson´s r	0.176	0.488	0.224	-0.245	-0.11	0.437	0.375	0.629	0.265	0.41		0.305	0.423
	p value	0.39	0.011	0.271	0.228	0.646	0.026	0.059	0.001	0.191	0.038		0.129	0.031
**OAS1**	Pearson´s r	0.341	0.257	0.107	-0.028	0.123	0.428	0.633	0.336	0.127	0.424	0.305		0.404
	p value	0.088	0.205	0.601	0.892	0.604	0.029	0.001	0.094	0.536	0.031	0.129		0.04
**STAT1**	Pearson´s r	0.253	0.417	0.136	-0.037	0.065	-0.002	0.62	0.394	0.374	0.266	0.423	0.404	
	p value	0.212	0.034	0.508	0.856	0.785	0.993	0.001	0.046	0.06	0.188	0.031	0.04	

		ATF4	ATF5	CHOP	GDF15	FGF21	PGC1α	TRAP1	DRP1	FIS1	OPA1	NFκB	OAS1	STAT1
ATF4	Correlación de Pearson		0.804	0.655	0.278	0.400	0.036	0.299	0.246	0.509	0.479	0.176	0.341	0.253
	Sig. (bilateral)		<0.001	<0.001	0.169	0.080	0.862	0.138	0.227	0.008	0.013	0.390	0.088	0.212
ATF5	Correlación de Pearson	0.804		0.514	-0.045	0.279	-0.048	0.240	0.373	0.709	0.560	0.488	0.257	0.417
	Sig. (bilateral)	<0.001		0.007	0.828	0.234	0.816	0.238	0.060	<0.001	0.003	0.011	0.205	0.034
CHOP	Correlación de Pearson	0.655	0.514		0.225	0.197	0.167	0.229	0.343	0.475	0.442	0.224	0.107	0.136
	Sig. (bilateral)	<0.001	0.007		0.270	0.405	0.416	0.261	0.086	0.014	0.024	0.271	0.601	0.508
GDF15	Correlación de Pearson	0.278	-0.045	0.225		-0.076	-0.099	0.155	-0.140	0.102	-0.069	-0.245	-0.028	-0.037
	Sig. (bilateral)	0.169	0.828	0.270		0.750	0.630	0.449	0.494	0.621	0.736	0.228	0.892	0.856
FGF21	Correlación de Pearson	0.400	0.279	0.197	-0.076		-0.217	-0.179	-0.115	0.314	-0.058	-0.110	0.123	0.065
	Sig. (bilateral)	0.080	0.234	0.405	0.750		0.359	0.450	0.630	0.177	0.809	0.646	0.604	0.785
PGC1a	Correlación de Pearson	0.036	-0.048	0.167	-0.099	-0.217		0.418	0.523	-0.040	0.479	0.437	0.428	-0.002
	Sig. (bilateral)	0.862	0.816	0.416	0.630	0.359		0.034	0.006	0.847	0.013	0.026	0.029	0.993
TRAP1	Correlación de Pearson	0.299	0.240	0.229	0.155	-0.179	0.418		0.440	0.316	0.305	0.375	0.633	0.620
	Sig. (bilateral)	0.138	0.238	0.261	0.449	0.450	0.034		0.024	0.116	0.130	0.059	0.001	0.001
DRP1	Correlación de Pearson	0.246	0.373	0.343	-0.140	-0.115	0.523	0.440		0.286	0.538	0.629	0.336	0.394

	Sig. (bilateral)	0.227	0.060	0.086	0.494	0.630	0.006	0.024		0.156	0.005	0.001	0.094	0.046
FIS1	Correlación de Pearson	0.509	0.709	0.475	0.102	0.314	-0.040	0.316	0.286		0.388	0.265	0.127	0.374
	Sig. (bilateral)	0.008	<0.001	0.014	0.621	0.177	0.847	0.116	0.156		0.050	0.191	0.536	0.060
OPA1	Correlación de Pearson	0.479	0.560	0.442	-0.069	-0.058	0.479	0.305	0.538	0.388		0.410	0.424	0.266
	Sig. (bilateral)	0.013	0.003	0.024	0.736	0.809	0.013	0.130	0.005	0.050		0.038	0.031	0.188
NFκB	Correlación de Pearson	0.176	0.488	0.224	-0.245	-0.110	0.437	0.375	0.629	0.265	0.410		0.305	0.423
	Sig. (bilateral)	0.390	0.011	0.271	0.228	0.646	0.026	0.059	0.001	0.191	0.038		0.129	0.031
OAS1	Correlación de Pearson	0.341	0.257	0.107	-0.028	0.123	0.428	0.633	0.336	0.127	0.424	0.305		0.404
	Sig. (bilateral)	0.088	0.205	0.601	0.892	0.604	0.029	0.001	0.094	0.536	0.031	0.129		0.040
STAT1	Correlación de Pearson	0.253	0.417	0.136	-0.037	0.065	-0.002	0.620	0.394	0.374	0.266	0.423	0.404	
	Sig. (bilateral)	0.212	0.034	0.508	0.856	0.785	0.993	0.001	0.046	0.060	0.188	0.031	0.040	
