# Supplementary figures and images for: Mitochondrial Stress Links Environmental Triggers with Pro-Inflammatory Signaling in Crohn’s Disease
Source: Antioxidants (Basel). 2023 Dec 13;12(12):2105. doi: 10.3390/antiox12122105 (PMC10741078; doi:10.3390/antiox12122105)

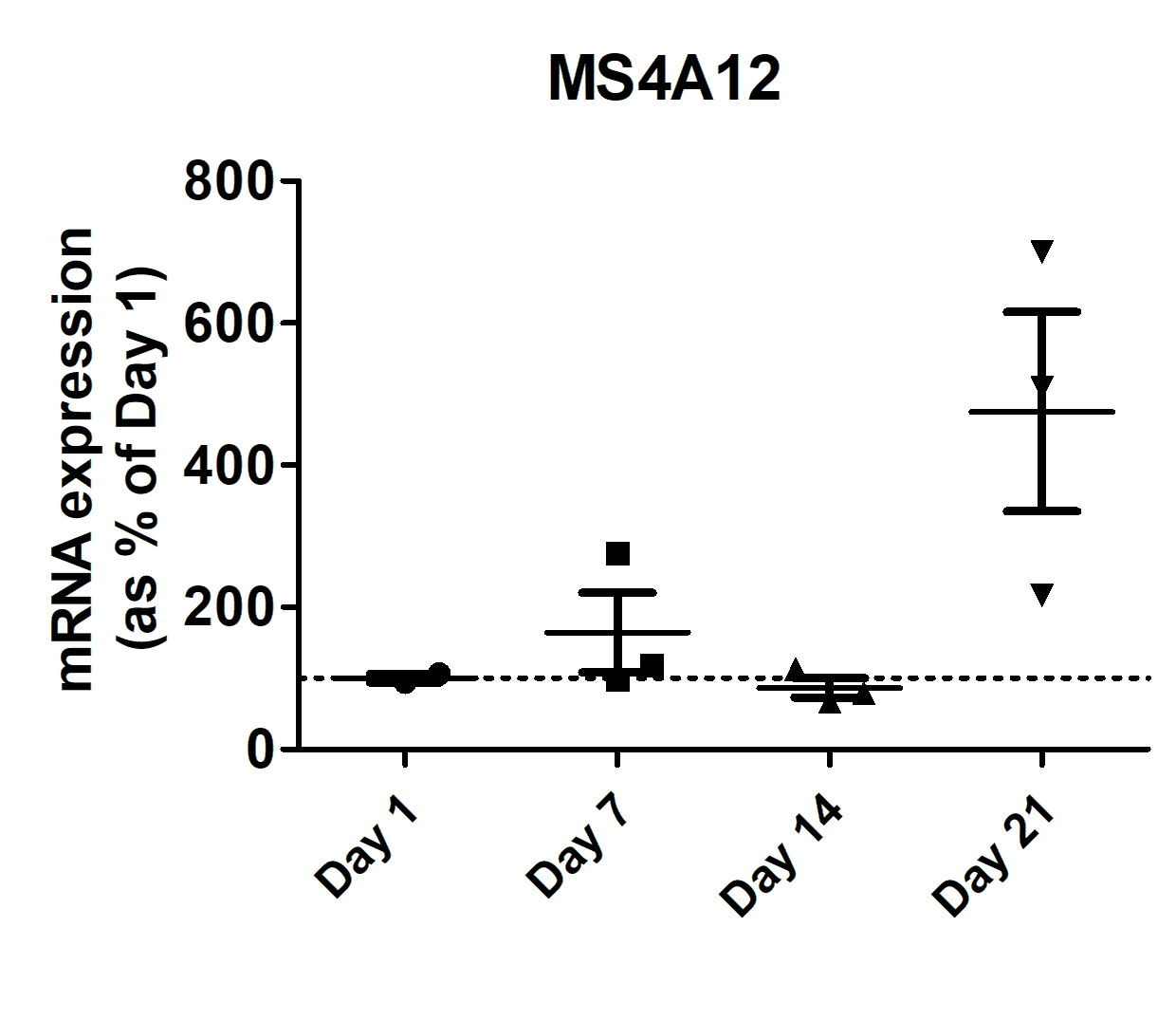

Supplement: Supplementary file 1 [file antioxidants-12-02105-s001.zip › Supplemental Figure S1.tif]

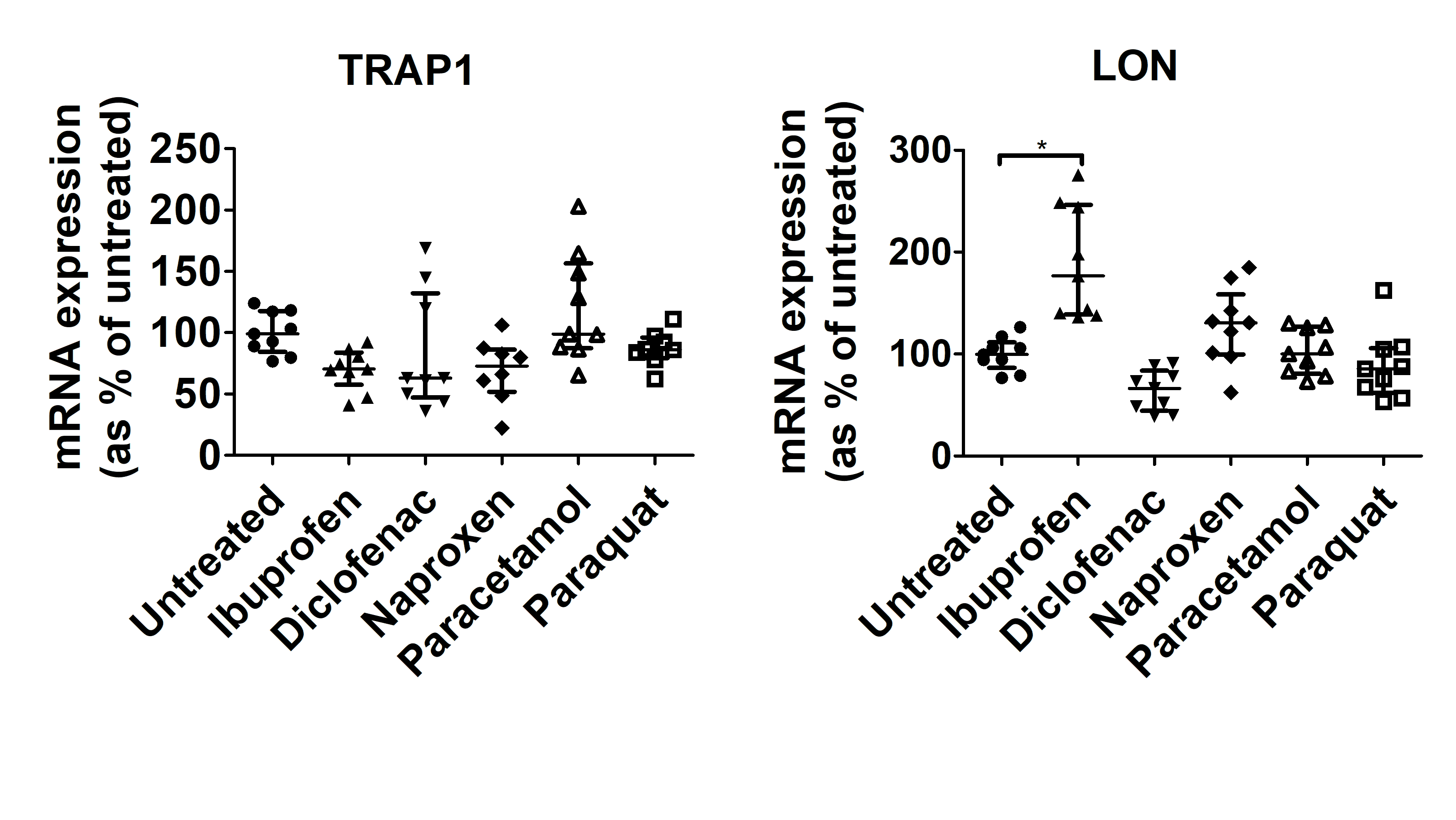

Supplement: Supplementary file 1 [file antioxidants-12-02105-s001.zip › Supplemental Figure S2.tif]

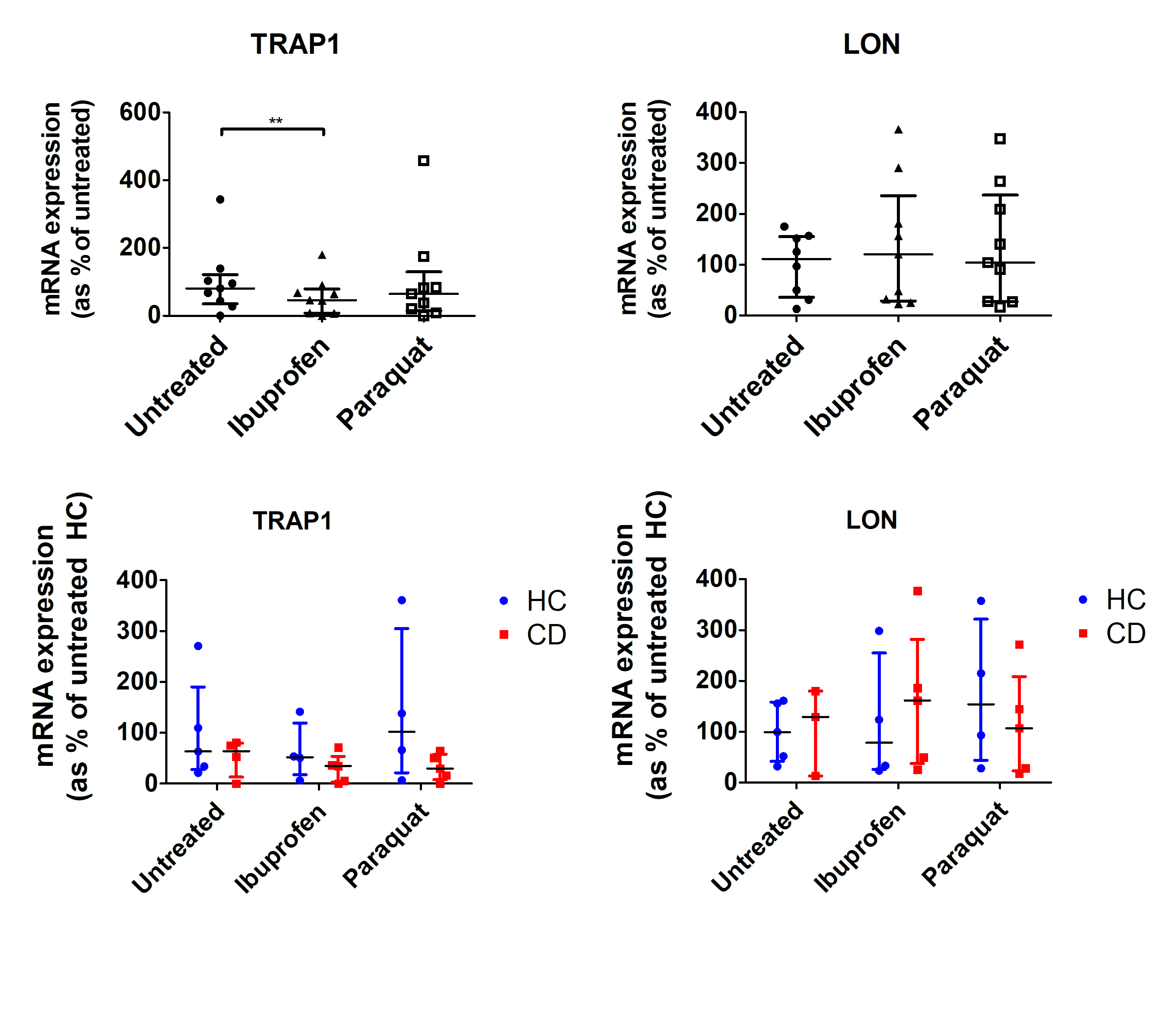

Supplement: Supplementary file 1 [file antioxidants-12-02105-s001.zip › Supplemental Figure S3.tif]

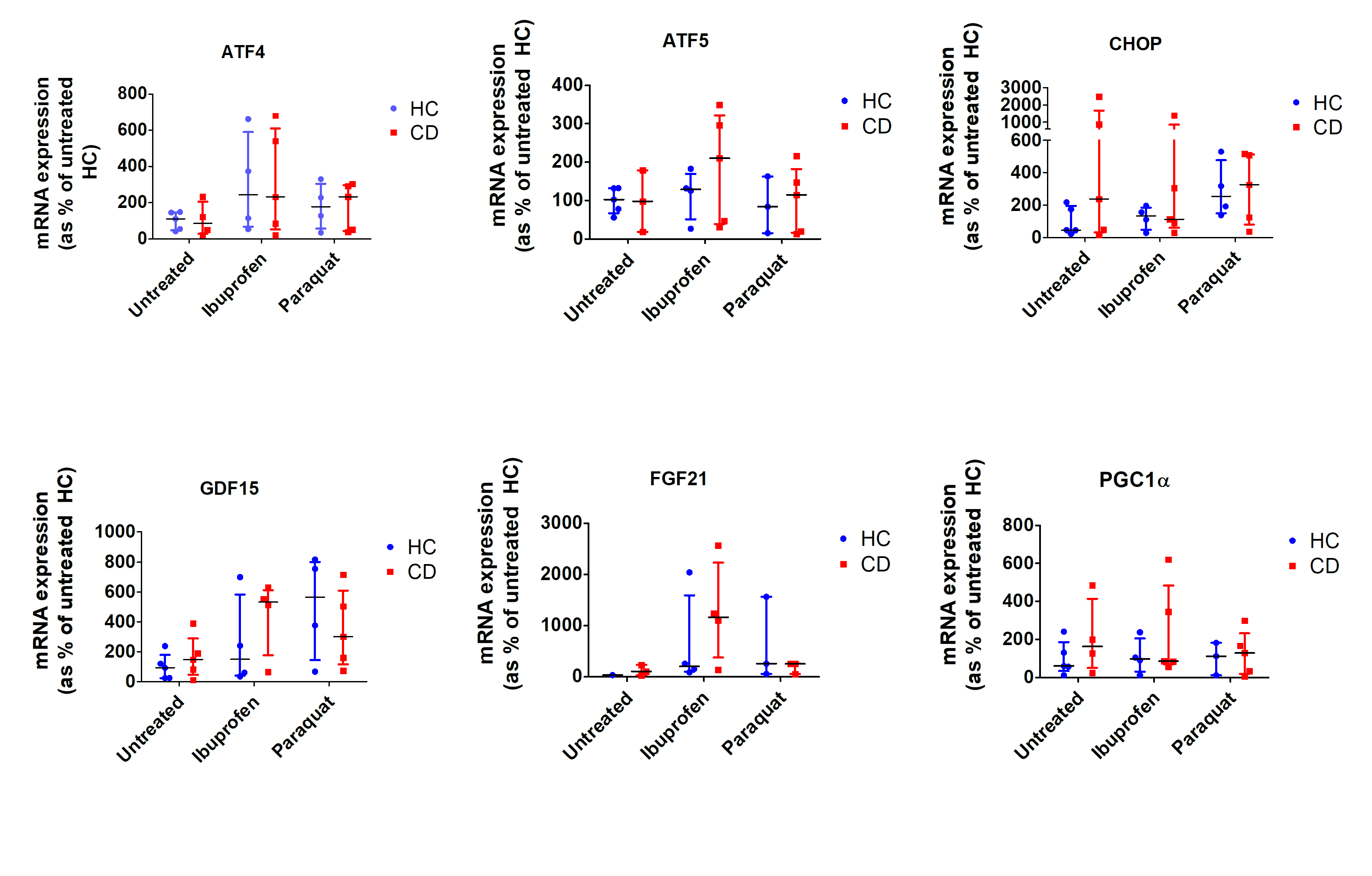

Supplement: Supplementary file 1 [file antioxidants-12-02105-s001.zip › Supplemental Figure S4.tif]

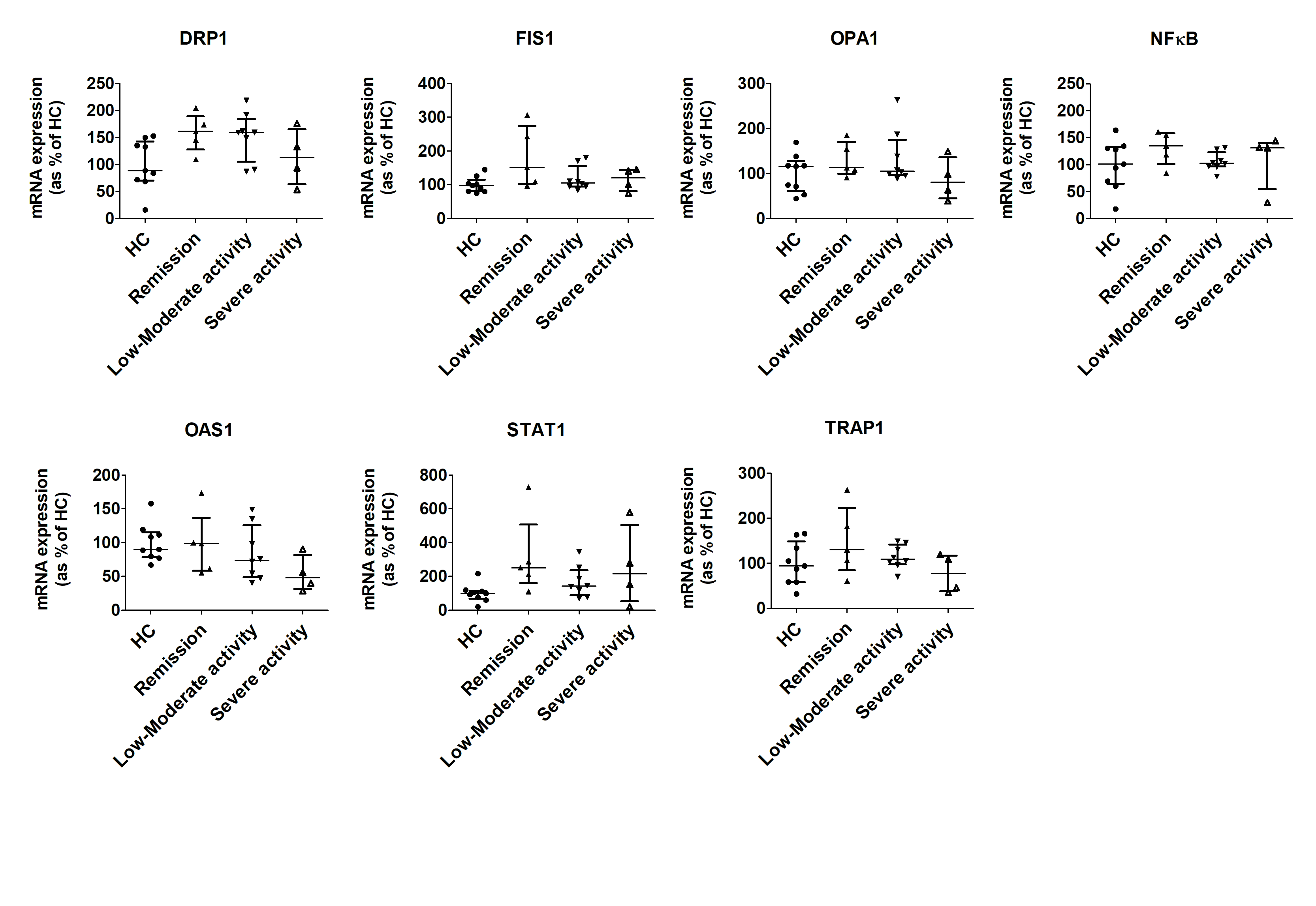

Supplement: Supplementary file 1 [file antioxidants-12-02105-s001.zip › Supplemental Figure S5.tif]
